# Pickering emulsion technology based on the concept of “the combination of medicine and adjuvant” to enhance the oxidation stability of volatile oils in solid preparations—taking Lingzhu Pulvis as an example†

**DOI:** 10.1039/d2ra04433a

**Published:** 2022-09-27

**Authors:** Lei Peng, Xiao-Fei Zhang, Dong-Yan Guo, Bing-Tao Zhai, Mei Wang, Jun-Bo Zou, Ya-Jun Shi

**Affiliations:** Shaanxi Province Key Laboratory of New Drugs and Chinese Medicine Foundation Research, Pharmacy College, Shaanxi University of Chinese Medicine Xianyang 712046 China 2051078@sntcm.edu.cn 2051004@sntcm.edu.cn

## Abstract

The antioxidant properties of the volatile oil of *Acorus calamus* in Lingzhu Pulvis may be enhanced by the introduction of Pickering emulsion technology based on the concept of “the combination of medicine and adjuvant”. The characterization of each drinking tablet of Lingzhu Pulvis was conducted to determine the stabilizer. The optimal stabilizer concentration, oil–water ratio and preparation method of the Pickering emulsion were then determined and analyzed using NIR. The contents of malondialdehyde and peroxide in the volatile oils of each group were compared at different AIBA concentrations. The trends of the components were then analyzed by GC-MS. The pearl powder was screened as the stabilizer of the Pickering emulsion; the pearl powder concentration of 0.065 g mL^−1^ and the oil–water ratio of 9 : 11 were found to be the optimal emulsion formation conditions, and the high-pressure homogenization method was the optimal preparation method. The NIR analysis showed that the volatile oil was wrapped by the pearl powder and no new chemical structure formed in the Pickering emulsion. The Pickering emulsions had lower oxidation levels than the crude oil groups at AIBA concentrations of 5, 10, and 15 mg mL^−1^. The results of the GC-MS analysis showed that the antioxidant properties of the volatile components were significantly higher in the Pickering emulsion group compared to the crude oil group. Pickering emulsions can be used to enhance the antioxidant properties of volatile components in oil-containing solid formulations based on the concept of “the combination of medicine and adjuvant”.

## Abstract 2

The antioxidant properties of the volatile oil of *Acorus calamus* in Lingzhu Pulvis may be enhanced by the introduction of Pickering emulsion technology based on the concept of “the combination of medicine and adjuvant”.

## Graphical abstract

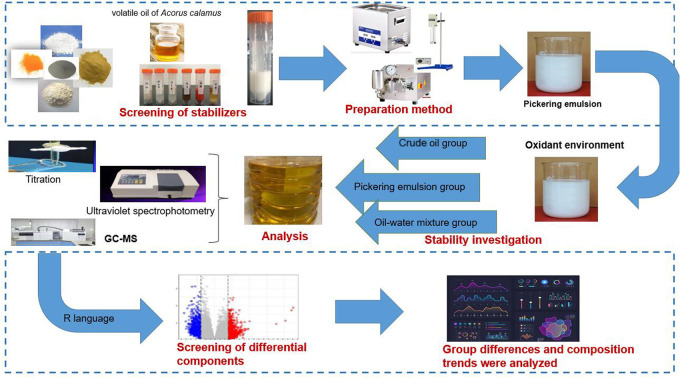

## 1 Introduction

Chinese volatile oils have many applications in clinical and cosmetic fields because of their numerous changes pharmacological effects, to the as opening the orifices, relieving pain, and dispersing pathogen.^1^ However, the defects of volatile oils used in Chinese medicine, such as easy to oxidize and difficulty in preserving the preparations, limit their application.^2^ The main methods for the improvement of these properties are the β-cyclodextrin encapsulation technique and liposome preparation, which have been studied in many fields and partially applied in practice. However, they also have some problems, such as the introduction of exogenous excipients, disruption of the original activity of the volatile oil during the preparation process, sensitivity to the environment, and biosafety, which should be examined.

In the eighteenth century, colloidal particles were first discovered and used as emulsifiers, and in the following years, Pickering began research on solid particles as stabilizers for emulsions that these emulsions are also called Pickering emulsions.^3^ Pickering emulsions have been widely used in food, cosmetics, and pharmaceuticals in recent years because of their environment-friendly and stable properties.^4^ Compared with traditional emulsions, they have higher stability, low toxicity, biocompatibility as well as broad application prospects for the improvement of the stability of volatile oils used in traditional Chinese medicine. The current applications of Pickering emulsions in Chinese herbal volatile oils are mainly to improve the dispersion of Chinese herbal volatile oils, antibacterial effects, stability, and other areas.^5,6^

In Chinese medicine preparations, “the combination of medicine and adjuvant” is an important principle for the use of excipients and also a distinctive feature of Chinese medicine preparations different from chemical preparations.^7^ In this hypothesis, from the therapeutic point of view, the powder acts as a stabilizer to improve the stability of the volatile oil, which in turn ensures the clinical efficacy and stability of the formulation, reflecting the characteristics of “auxiliary medicine”. From the formulation point of view, the use of the special properties of the prescription powder, which acts as a stabilizer, to prepare the volatile oil solid particle emulsion is the embodiment of “medicine for the auxiliary”.

Lingzhu Pulvis is a century-old prescription produced by Lei Yunsheng and has been passed on as a national and intangible cultural heritage. It has antipyretic, sedative, and tranquilizing effects and is mostly used clinically for children with fever, restlessness and cough with phlegm.^8^ The prescription contains different types of powder for animal medicine (*Cornu antelopis*, artificial bezoar), plant medicine (calamus), mineral medicine (pearl powder, cinnabar), extracts (*Bombyx batryticatus*, Dan Nanxing extract), and resin (amber), which basically cover the protein, cellulose, mineral, starch, and polysaccharide stabilizers used in the preparation of solid particle emulsions. In the preparation process of Lingzhu Pulvis, the pearl powder accounts for 7.5% of the whole formula, and the volatile oil of *Acorus calamus* accounts for 1%. The amount of pearl powder used to prepare the Pickering emulsion of the volatile oil of *Acorus calamus* according to this experimental method is very small, less than one-thousandth of the whole formula, which is negligible. It is an ideal research object for the implementation of the idea of “the combination of medicine and adjuvant”. In this study, we introduced the Pickering emulsion technology to improve the antioxidant properties of the volatile oil of *Acorus calamus* based on the idea of “the combination of medicine and adjuvant”, which provides a research basis for the improvement of the stability of Lingzhu Pulvis and other solid formulations containing oils in future.

## 2 Materials and methods

### 2.1 Material

The equipment used were as follows: MH-3000 temperature-regulating electric heating jacket (Beijing Kewei Yongxing Instrument Co., Ltd.), electric heating blast dryer (Shanghai-Heng Scientific Instruments Co., Ltd.), HG-3 magnetic heating stirrer (Changzhou Guohua Electric Co., Ltd.), JY-3002 one-hundred-thousandth analytical balance (Shanghai Puchun Instrument Co., Ltd.), IKA T18 digital high-speed disperser (Shanghai Tucson Vision Technology Co., Ltd.), AH-BASIC high pressure homogenizer (Antos Nano Technology Co., Ltd.), DM3000 biological microscope (Leica Microsystems Trading Co., Ltd.), TENSOR-27 Fourier transform infrared spectrometer (Bruker, Germany), ANTIRIS II Fourier transform near-infrared spectrometer (Thermo Fisher Scientific, USA), K100C-KRUSS automatic surface tension and contact angle tester (KRUSS, Germany), UV spectrophotometer (Shanghai Yoke Instrument Co., Ltd.), Zeiss Sigma300 (Carl Zeiss (Shanghai) Management Co., Ltd.), nanoparticle size potential analyzer (ZS90, Malvern Instruments Ltd., UK), acid burette, and Kjeldahl flask.

The reagents used were as follows: the volatile oil of *Acorus calamus*, pearl powder, artificial bezoar, amber, cinnabar, bilberry and Dan Nanxing extract from Lei Yun Shang Pharmaceutical Group Co., Ltd, chloroform (Lot No. 20200203, Tianjin Tianli Chemical Reagent Co., Ltd.), 2-thiobarbituric acid (TBA, Shanghai Kefeng Industrial Co., Ltd., Lot No. 20170921), trichloroacetic acid, and 1,1,3,3-tetraethoxypropane (Shanghai Maclean Biochemical Technology Co., Ltd., Lot C10050555, purity ≥ 97%). The water was distilled and the rest of the reagents were analytically pure.

### 2.2 Method

Characterization and screening of the stabilizer powder properties.

#### 2.2.1 Solubility

Solubility: using the JY-3002 one-hundred-thousandth analytical balance, 0.01 g each of pearl powder, amber, cinnabar, artificial bezoar, *Cornu antelopis*, and the mixture of *Bombyx batryticatus* and Arisaema cum Bile was weighed, added in 100 mL of water, respectively, and shaken strongly for 30 s every 5 min; the dissolution state was observed for 30 min.

#### 2.2.2 Microstructure

Trace samples were glued directly to the conductive adhesive and sprayed with gold for 45 s using an Oxford Quorum SC7620 sputter coater at 10 mA; the sample morphology was subsequently captured using a ZEISS Sigma 300 scanning electron microscope.

#### 2.2.3 Preparation of emulsions

An appropriate amount of the volatile oil of *Acorus calamus* was measured and mixed with water in a beaker, making a total of 6 parts, respectively. To this, an appropriate amount of pearl powder, amber, cinnabar, artificial cowry, artificial bezoar, *Cornu antelopis*, or *Bombyx batryticatus* and Arisaema cum Bile was added using a high-speed dispersion machine to form an emulsion; the emulsion phase was separated, and the emulsion layer color was observed.

#### 2.2.4 Staining method

The emulsion samples were applied to the slides and stained once each with Sudan-III and methylene blue, and the staining results were observed.

#### 2.2.5 Determination of zeta potential

The zeta potential of each sample was analyzed using a Malvern nanoparticle sizer. Each sample was run three times in parallel at 25 °C.

#### 2.2.6 Preferred concentration of the stabilizer

To 14 mL of water and 6 mL of the volatile oil of *Acorus calamus*, which made a total of 16 parts, different amounts of pearl powder between 0.1 and 1.6 g were added with an increment of 0.1 g in each sample, respectively, and emulsified using a high-speed disperser. The optimal Pickering emulsion concentration was determined by observing the amount of oil encapsulation and emulsion formation.

#### 2.2.7 Preferred oil–water ratio

To five 45 mL centrifuge tubes containing 6 mL of the volatile oil of *Acorus calamus* each, 14 mL, 11.14 mL, 9 mL, 7.33 mL, and 6 mL of distilled water, as well as 1.30 g, 1.11 g, 0.96 g, 0.87 g, and 0.78 g of pearl powder were added, respectively and a high-speed disperser was used to form emulsions. The amount of emulsion formed and the amount of oil coating was observed to determine the optimal oil–water ratio.

#### 2.2.8 Preparation method

To three 45 mL centrifuge tubes, 6 mL of the volatile oil of *Acorus calamus*, 14 mL distilled water and 1.3 g pearl powder were added and mixed using a magnetic stirrer, high-speed disperser and high-pressure homogenizer, respectively. The Pickering emulsion morphology was observed using a DM3000 biomicroscope to determine the optimal preparation process.

#### 2.2.9 Near-infrared spectral characterization

Appropriate amounts of the volatile oil of *Acorus calamus*, pearl powder suspension and Pickering emulsion samples were placed in a 2 mm quartz cuvette for spectral acquisition in the range of 4000–1000 cm^−1^ at room temperature, a scan number of 32, a resolution of 8 cm^−1^ and a gain of 1×. The spectra were collected with the air as the reference minus the background, and each sample was repeated three times to obtain the average NIR spectra.

### 2.3 Stability experiments

#### 2.3.1 Collection of volatile oils in oxidant stability experiments

The crude oil, oil–water mixture with a 45% oil ratio and Pickering emulsion were placed in 100 mL evaporation dishes, and AIBA was added to make their concentrations 5, 10 and 15 mg mL^−1^, respectively. After 30 min, the oil–water mixture and Pickering emulsion were distilled for 8 h and separated by water vapor distillation. Three parallel sets of experiments were performed.

#### 2.3.2 Determination of malondialdehyde substances in the volatile oil

##### 2.3.2.1 Determination of the standard curve

For this, 0.01, 0.02, 0.03, 0.04, 0.05, 0.06, 0.07, and 0.08 mL of the malondialdehyde standard solution (0.315 g of 1,1,3,3-tetraethoxypropane dissolved in water and diluted to 1000 mL) was accurately pipetted into 10 mL volumetric flasks, and the volume was made up with distilled water to 10.00 mL to obtain the standard concentrations of 0.01, 0.02, 0.03, 0.04, 0.05, 0.06, 0.07, 0.08 μg mL^−1^ malondialdehyde. To these, 5 mL of thiobarbituric acid (TBA) solution (0.288 g thiobarbituric acid diluted to 100 mL with water, 0.02 mol L^−1^) was added, mixed well, heated in a water bath at 90 °C for 40 min, and then removed. Then, 5.00 mL of chloroform was added after cooling, shaken well, and let to stand for 1 h. The absorbance of the supernatants was measured at 532 nm, and the standard curve was plotted with the mass concentrations of the standard solutions as the horizontal coordinates and the absorbance as the vertical coordinates.

##### 2.3.2.2 Sample preparation and determination

500 μL of the above volatile oil samples were taken in a 10 mL volumetric flask, and a mixed solution of trichloroacetic acid (37.5 g trichloroacetic acid and 0.50 g sodium ethylenediaminetetraacetate, dissolved in water and diluted to 500 mL) was fixed to the scale, sonicated for 10 min, and filtered. Then, 5.00 mL of the filtrate was taken in a conical flask, 5.00 mL of TBA solution was added, heated at 90 °C for 4 h, and cooled away from light for 1 h. Later, 5.00 mL of chloroform was added, and the mixture was shaken thoroughly and left for 1 h. The absorbance of the supernatant was measured at 532 nm, and the malondialdehyde content was calculated.

#### 2.3.3 Determination of the peroxide value of the volatile oil

Separately, 500 μL of the above volatile oil samples were taken in centrifuge tubes. Using 10 mL of a trichloromethane–glacial acetic acid mixture (v_1_/v_2_ = 4 : 6), these samples were washed into corked conical flasks. To this, 1 mL saturated potassium iodide solution was accurately added, tightly plugged, shaken for 0.5 min, and placed in the dark for 3 min. Then, 30 mL of water and 1 mL of 1% starch indicator were added and titrated with 0.001 mol L^−1^ sodium thiosulfate standard solution (26 g sodium thiosulfate and 0.20 g anhydrous sodium carbonate dissolved in 1000 mL water, boiled slowly for 10 min, and diluted after cooling) until the solution disappeared, which was considered the endpoint; the volume of consumed sodium thiosulfate standard solution was used to calculate the POV value.

#### 2.3.4 Analysis of the volatile oil components by gas chromatography-mass spectrometry

##### 2.3.4.1 Determination of the volatile oil composition by GC-MS after intervention under different conditions

###### GC-MS conditions

HP-5MS quartz capillary column (30 m × 0.25 mm, 0.25 μm), helium (99.999% purity) as the carrier gas, flow rate 1 mL min^−1^; injection volume 1 μL; shunt ratio 10 : 1; inlet temperature 230 °C; temperature program: 50 °C for 2 min, to 110 °C for 2 min at 5 °C min^−1^, to 120 °C for 5 min at 2 °C min^−1^, to 125 °C for 10 min at 0.5 °C min^−1^, to 200 °C for 2 min at 4 °C min^−1^, to 250 °C for 2 min at 10 °C min^−1^. Mass spectrometry conditions: ionization mode EI, ion mode ESI, electron energy of 70 eV, quadrupole temperature 150 °C, ion source temperature 230 °C, scan range *m*/*z* 35–500, solvent delay 3 min.

###### Sample solution preparation

100 μL of the samples mentioned above in 2.3.1 were taken in 10 mL brown volumetric flasks, respectively, and the volume was fixed with ether, then, an appropriate amount of anhydrous sodium sulfate was added, after which 2 mL of each sample was precisely weighed, filtered through a membrane in liquid-phase vials, and used for GC-MS for composition determination.

##### 2.3.4.2 Screening of differential components

After the GC-MS data acquisition was completed, the components were characterized by the Agilent database analysis software using the NIST 14.0 database; 27 sets of data were summarized and screened for differential components at different AIBA concentrations using the R language limma package. Moreover, the heat diagram, PCA plots, concentration change maps and box plots were drawn using the differential components after de-weighting.

## 3 Result and discussion

### 3.1 Characterization and screening of the stabilizer powder properties

**Fig. 1:**
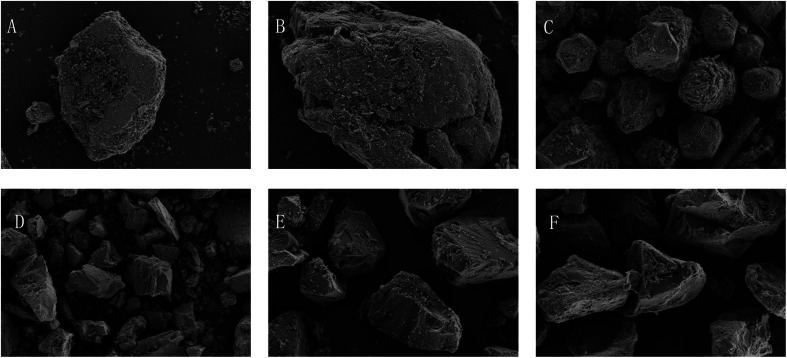
Microscopic morphology of each sample particle (×1000). (A) Pearl powder; (B) *Cornu antelopis*; (C) artificial bezoar; (D) cinnabar; (E) amber; (F) *Bombyx batryticatus* and Arisaema cum Bile.

**Fig. 2:**
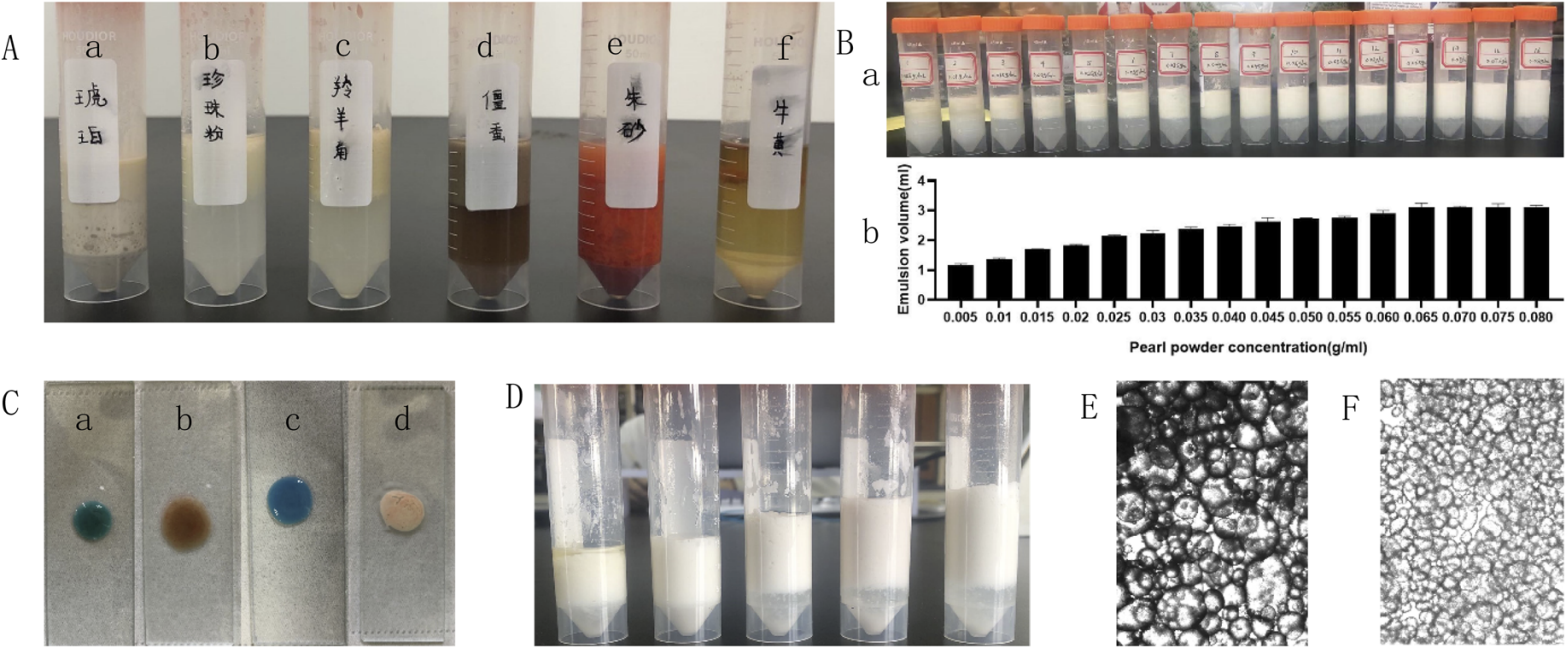
The preparation methods of the emulsions of various medicinal materials and identification of their emulsion types. (A) Emulsion-forming state ((a) amber; (b) pearl powder; (c) *Cornu antelopis*; (d) *Bombyx batryticatus* and Arisaema cum Bile; (e) cinnabar; (f) artificial bezoar); (B) emulsion volume at different pearl powder concentrations; (C) staining method to identify each herb into emulsion types ((a) *Cornu antelopis*; (b) *Bombyx batryticatus* and Arisaema cum Bile; (c) pearl powder; (d) amber); (D) emulsion state at different oil–water ratios; (E) the Pickering emulsion prepared by the high-speed dispersion method (×100); (F) the Pickering emulsion prepared by the high-pressure homogenization method (×100).

**Fig. 3:**
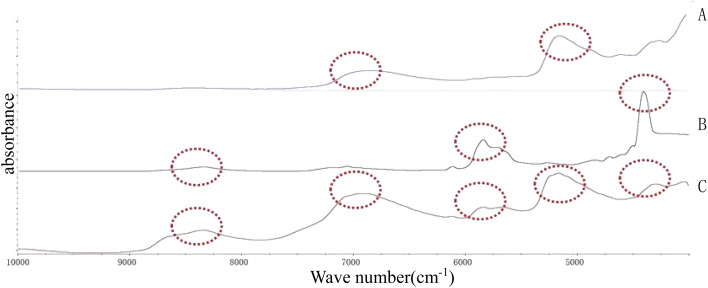
Near-infrared spectrogram. (A) Pearl powder suspension; (B) the volatile oil of *Acorus calamus*; (C) Pickering emulsion.

The results are shown in Table 1. All the tablets were insoluble, and their dissolution in the water while forming emulsions could be neglected. From Fig. 1, it can be seen that the pearl powder formed the shape of large diamond-shaped lumps, *Cornu antelopis* was in the shape of raindrops with gaps on the surface, and artificial bezoar presented small round lump rows, while cinnabar, amber, *Bombyx batryticatus* and Arisaema cum Bile existed as small conical lumps. From Fig. 2A, it can be seen that artificial bezoar and cinnabar could not form emulsions, while amber, as well as *Bombyx batryticatus* and Arisaema cum Bile, formed W/O emulsions (water-in-oil emulsion), as shown in Fig. 2C. On comparing the zeta potentials of the *Cornu antelopis* and pearl powder emulsions, the absolute zeta potential value of the pearl powder emulsion was greater and more stable, and hence, pearl powder was chosen as the suitable stabilizer for the Pickering emulsion. With the increment of pearl powder concentration, the emulsion layer formed gradually increased, as shown in Fig. 2B. After the concentration exceeded 0.065 g mL^−1^, the emulsion volume stabilized, and the formed emulsion layer stratified with an increase in time. Thus, the maximum emulsion volume without stratification was 0.065 g mL^−1^. Next, the emulsion state under different oil–water ratios was observed; the emulsion layer and water layer delamination appeared at 30%, 35% and 40% oil ratios, while the emulsion layer and oil layer delamination appeared at 50% oil ratio, and the emulsion increased in stability without delamination at 45% oil ratio, as shown in Fig. 2D. The oil ratio of 45% was selected for the formation of the Pickering emulsion as the optimal oil–water ratio. The solution with 45% oil and 0.065 g mL^−1^ pearl powder was emulsified by using a magnetic stirrer, high-speed disperser and high-pressure homogenizer, respectively, and the emulsion-forming state was compared between the three different processes. It was seen that the magnetic stirring method did not form an emulsion, and then, the DM3000 biological microscope was used to observe emulsion formation by the high-speed disperser and high-pressure homogenization methods. The best emulsion formation was achieved by the high-pressure homogenization method, as shown in Fig. 2E and F. According to Fig. 3, the infrared characteristic spectra of the Pickering emulsion and the suspensions of the volatile oil of *Acorus calamus* and pearl powder were extremely similar; the peak types and peak numbers were basically the same, and no new absorption peaks were generated in the modified dispersion. Therefore, the introduction of Pickering emulsion technology did not destroy the original components, and no new components were generated.

**Table 1:** Characterization and emulsion type of each stabilizing agent. Note: O/W: oil-in-water emulsions, an emulsification system that disperses oil in water, with oil as the inner phase and water as the continuous outer phase; W/O: water-in-oil emulsion, an emulsification system that disperses water in oil, with water as the inner phase and oil as the continuous outer phase

Samples	Solubility	Emulsion type	Zeta potential
Pearl powder	Insoluble	O/W	−2.72 ± 0.41
Amber	Insoluble	W/O	—
Cinnabar	Insoluble	—	—
Artificial bezoar	Insoluble	—	—
*Cornu antelopis*	Insoluble	O/W	0.74 ± 0.21
*Bombyx batryticatus* and Arisaema cum Bile	Insoluble	W/O	—

In terms of solubility, all types of tablets in antelope Zhu San can be used to prepare Pickering emulsion. The observation of their emulsion-forming states showed that artificial bezoar and cinnabar could not form emulsions. The emulsion structures of amber and *Bombyx batryticatus* and Arisaema cum Bile were W/O, which is not consistent with the purpose of this experiment. The emulsion structures formed by *Cornu antelopis* and pearl powder were consistent with the purpose of this experiment. Further, the comparison of the zeta potentials of the two showed that pearl powder was more stable, and finally, pearl powder was chosen as the preferred stabilizer.

As seen in the figure, pearl powder, *Cornu antelopis*, artificial bezoar, cinnabar, amber, and *Bombyx batryticatus* and Arisaema cum Bile were irregularly shaped lumpy powders. Pearl powder had a smaller particle size and more uniform appearance compared with the antelope horn, which is presumed to be the reason why the emulsion formed by pearl powder was more stable than that formed using the antelope horn.

In Fig. 2A, we can see the state of various powders in the emulsions, and Fig. 2C shows the identification of the categories of emulsions formed; Fig. 2B shows the emulsion states and volumes under different pearl powder concentrations, while Fig. 2D shows the emulsion state at different oil–water ratios. Moreover, in Fig. 2E and F, we can see that the Pickering emulsions prepared using the high-pressure homogenization method had smaller and more uniform particles. Based on these, the final pearl powder concentration, oil–water ratio, and preparation method to form the Pickering emulsion were determined.

From the figure, we can see that the absorption peaks of the Pickering emulsion were found in the spectra of both the volatile oil of *Acorus calamus* and pearl powder suspension, suggesting that the formed Pickering emulsion had not destroyed the original components, and no new components were generated.

### 3.2 Stability experiments

**Fig. 4:**
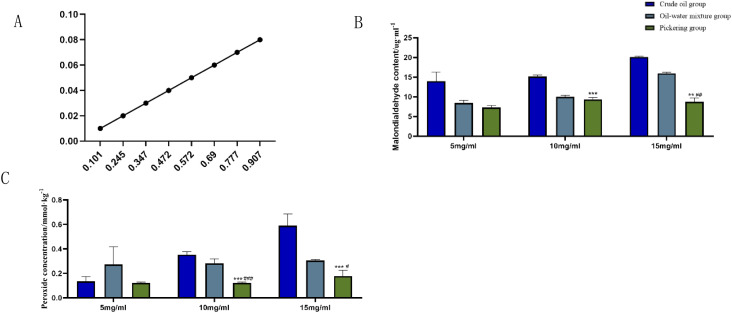
Standard curve of malondialdehyde and the comparison of this index among the groups. (A) The standard curve of malondialdehyde concentration and absorbance; (B) content of malondialdehyde at different AIBA concentrations; (C) content of peroxide at different AIBA concentrations. Compared with crude oil *P < 0.05, **P < 0.01, ***P < 0.001; compared with oil–water mixture group ^#^*P* < 0.05, ^##^*P* < 0.01, ^##^*P* < 0.001.

The standard curve of malondialdehyde concentration/absorbance was plotted using 1,1,3,3-tetraethoxypropane with a linear regression equation of *Y* = 0.0889*x* − 0.0007, *r* = 0.9989, as presented in Fig. 4A. Based on the standard curve, the malondialdehyde concentration in different samples was calculated. It can be seen that at the AIBA concentrations of 10 and 15 mg mL^−1^, the malondialdehyde content was significantly lower in the Pickering emulsion group compared with the crude oil group (*P* < 0.005, *P* < 0.001), as shown in Fig. 4B. At the AIBA concentration of 5 mg mL^−1^, there was no statistical difference between the crude oil and Pickering emulsion groups (*P* > 0.05), and at AIBA concentrations of 10 and 15 mg mL^−1^, the Pickering emulsion group had significantly lower peroxide content compared with the oil–water mixture and crude oil groups (*P* < 0.001), as shown in Fig. 4C.

It can be seen from the figure that the content of malondialdehyde and peroxide in both the crude oil and Pickering emulsion groups increased with the increase in oxidant concentration; however, the changes in malondialdehyde and peroxide in the Pickering emulsion were always lower than those in the crude oil group, and there was a significant difference between their contents in the two groups.

### 3.3 Analysis of the volatile oil components by gas chromatography-mass spectrometry

**Fig. 5:**
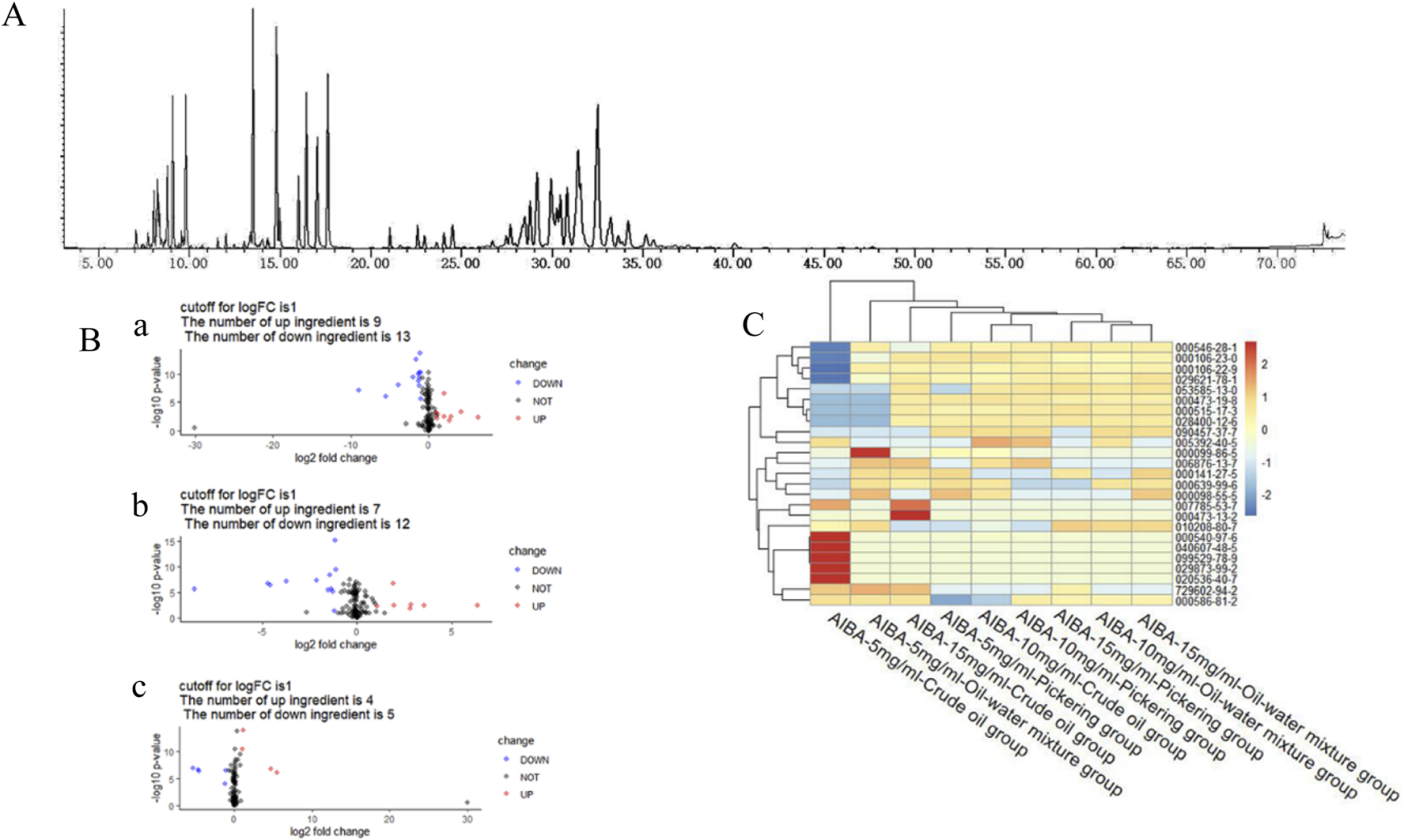
Total ion flow diagram of the volatile oil of *Acorus calamus*, volcano diagram of each AIBA concentration, and heat diagram of different components. (A) Total ion flow diagram of the volatile oil of *Acorus calamus*; (B) volcano diagram ((a) volcano diagram of 5 mg mL^−1^ and 10 mg mL^−1^; (b) volcano diagram of 5 mg mL^−1^ and 15 mg mL^−1^; (c) volcano diagram of 10 mg mL^−1^ and 15 mg mL^−1^); (C) heat diagram of the differential components.

**Fig. 6:**
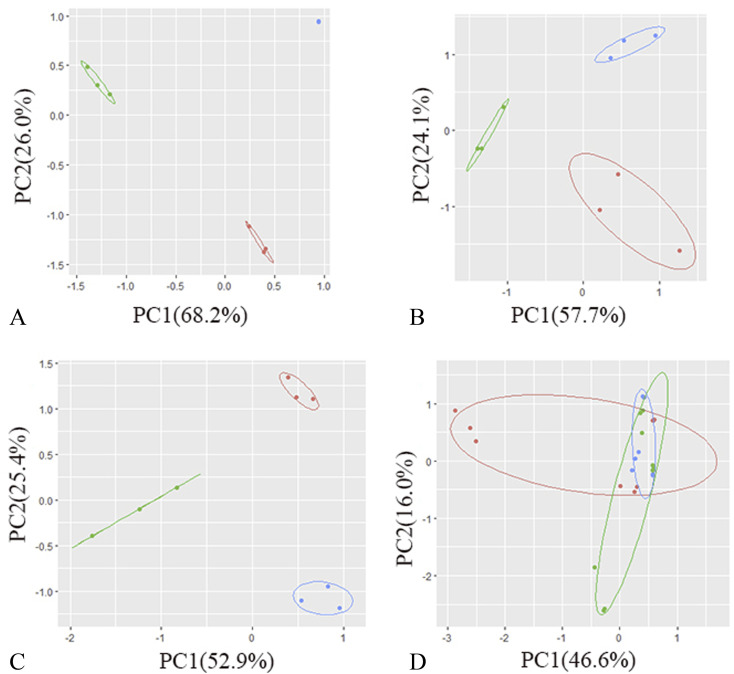
The PCA plots of the differential components. (A) PCA plots of the differential components in each group at 5 mg mL^−1^ AIBA; (B) PCA plots of the differential components in each group at 10 mg mL^−1^ AIBA; (C) PCA plots of the differential components in each group at 15 mg mL^−1^ AIBA; (D) PCA total plots of the differential components in each group. Red: oil–water mixture group; green: crude oil group; blue: Pickering group.

**Fig. 7:**
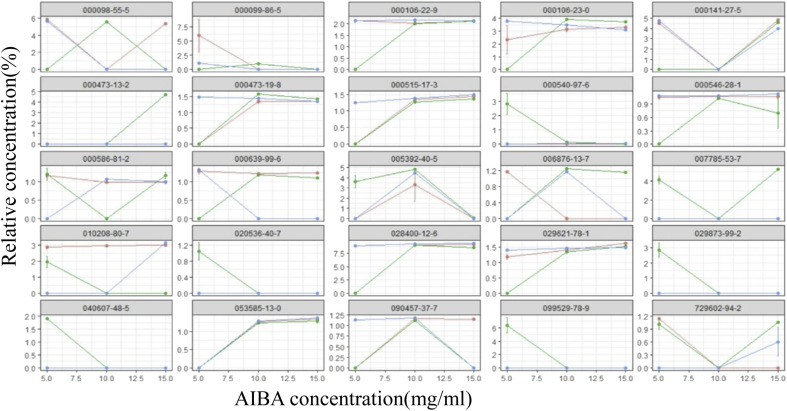
The differential component-concentration variation pattern of each group. Red: oil–water mixture group; green: crude oil group; blue: Pickering group.

**Fig. 8:**
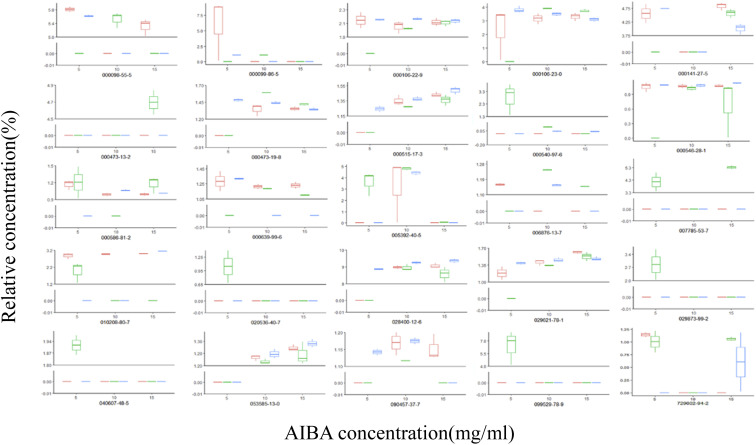
Box plots of the differential components for each group. Red: oil–water mixture group; green: crude oil group; blue: Pickering group.

The total ion flow diagram of the GC-MS analysis of the volatile oil of *Acorus calamus* is shown in Fig. 5A. It can be seen that the method could separate the chemical components in the volatile oil of *Acorus calamus* very well, and the summary of the components is shown in ESI Table 1.† Based on the volcano maps of the groups for each pair of AIBA concentrations, 22, 19 and 9 differential components were obtained, respectively, as shown in Fig. 5B, and a total of 25 differential components were obtained after de-weighting. The heat diagram and PCA plots of these 25 differential components between the different pairs of groups at AIBA concentrations of 5, 10, and 15 mg mL^−1^ were plotted separately, as shown in Fig. 5C, which could be divided into two groups according to the clustering information groups: ① AIBA-15 mg mL^−1^-oil–water mixture group, AIBA-10 mg mL^−1^-oil–water mixture group, AIBA-15 mg mL^−1^-Pickering group, AIBA-10 mg mL^−1^-Pickering group, AIBA-10 mg mL^−1^-crude oil group, AIBA-5 mg mL^−1^-Pickering group, AIBA-15 mg mL^−1^-crude oil group, AIBA-5 mg mL^−1^-oil–water mixture group. ② AIBA-5 mg mL^−1^-crude oil group. The varying components could be divided into two groups: ① beta-cedrene (000546-28-1), citronellal (000106-23-0), citronellol (000106-22-9), (−)-alpha-cuprenene (029621-78-1), (*E*)-gamma-bisabolene (053585-13-0), isocamphane (000473-19-8), selina-4(15),7(11)-diene (000515-17-3), alaskene (028400-12-6), spiro[4.5]dec-7-ene, 1,8-dimethyl-4-(1-methylethenyl)-(090457-37-7), citral (005392-40-5). ② Alpha-terpinene (000099-86-5), *cis*-pinane(006876-13-7), citral (000141-27-5), elemol (000639-99-6), alpha-terpineol (000098-55-5), (+)-alpha-terpineol (007785-53-7), alpha-selinene (000473-13-2), alpha-muurolene (010208-80-7), dodecamethyl cyclohexasiloxane (000540-97-6), 2-octen-1-ol, 3,7-dimethyl-(040607-48-5), (1*R*,5*R*)-1,8-dimethyl-4-(propan-2-ylidene)spiro[4.5]dec-7-ene (099529-78-9), 1-ethenyl-1-methyl-2-(1-methylethenyl)-4-(1-methylethylidene)-cyclohexane(029873-99-2), bicyclo[2.2.1]heptane, 2,2,3-trimethyl-, endo-(020536-40-7), (1*R*,4*R*,5*S*)-1,8-dimethyl-4-(prop-1-en-2-yl)spiro[4.5]dec-7-ene (729602-94-2), gamma-terpineol (000586-81-2). The results of the cluster analysis among the groups showed that the Pickering emulsion groups at low oxidant concentrations clustered with the crude oil group at high oxidant concentrations, indicating that the Pickering emulsion had a certain protective effect on the volatile oil of *Acorus calamus* present in it. The clustering data of the differential components are consistent with the trend of the differential components in Fig. 7 and 8. The PCA plots at different AIBA concentrations showed that the crude oil group, the oil–water mixture group and the Pickering emulsion group aggregated independently, as shown in Fig. 6A–C. The three points within the Pickering emulsion group were more aggregated and occupied a smaller range compared with those of the crude oil group, demonstrating that the repeatability and reproducibility of the Pickering emulsion group were better compared with that of the crude oil group. However, there was a partial overlap with the crude oil group in the total PCA plot, which is contrary to the results of PCA plots obtained with different AIBA concentrations. The possible reason is that the PC values occupy a lower percentage, and the mapping was not comprehensive in Fig. 6D. It can be seen from Fig. 7 that the content of the 25 compounds showed different trends with increasing oxidant concentration, but the trend was slowed down or reversed in the Pickering emulsion group compared to the crude oil group. Fig. 8 shows the higher reproducibility of the Pickering emulsion group compared with the crude oil group under the same experimental conditions. This result corroborates the conclusions drawn from the heat diagram and the PCA plots. Together, these findings show that the Pickering emulsion could improve the antioxidant properties of the volatile oil of *Acorus calamus*, which in turn served to improve the stability of the volatile oil of *Acorus calamus*. In addition, the 25 differential compounds showed different trends, and the decrease or increase in the content of some of their components at different oxidant concentrations may be related to the increase or decrease in the content of some of them, and the results provide some directions for further stability improvement in the future.

From Fig. 5A, it can be seen that this method can separate the chemical components of the volatile oil of *Acorus calamus* well; from the volcano diagram in Fig. 5B, the difference in compounds under different conditions can be observed; after de-weighting a total of 25 compounds, the two categories of clustering information obtained by plotting the heat diagram is presented in Fig. 5C.

It can be seen from Fig. 6A–C that the crude oil group and the Pickering emulsion group were well-separated in the PCA plot, and the circles of the Pickering emulsion group were smaller compared with those of the crude oil group. This indicates that the Pickering emulsion group differed from the crude oil group in its differential components and had higher stability and reproducibility, which are consistent with the results of the analysis of malondialdehyde and peroxide content. In Fig. 6D, the separation between the crude oil and Pickering emulsion groups is not as complete as in Fig. 6A–C. This may be due to the lower percentage of PC values that were not fully mapped.

It can be seen from the figure that different differential compounds in the crude oil group presented different trends at different oxidant concentrations, and some of their contents increased with increasing oxidant concentration, such as 000106-23-0, 000141-27-5, and 000473-13-2. A decreasing trend in content with the increase in the concentration of the oxidant was observed for some compounds, such as 000540-97-6, 010208-80-7, and 020536-40-7, *etc.* Those that did not show any trend of change with increasing oxidant concentration were 00098-55-5, 000473-19-8, 000546-28-1, *etc.* The different trends of the 25 compounds were slowed down or reversed in the Pickering emulsion, which is consistent with the results of the malondialdehyde and peroxide content, heat diagram and PCA plots.

From the figure, it can be seen that the trends of change observed in this analysis are consistent with Fig. 7. In addition, the box plots reveal that compared with the crude oil group, the experimental reproducibility of each differential component in the Pickering emulsion group at different oxidant concentrations was better, which further indicates the protective effect of the Pickering emulsion on the volatile oil of *Acorus calamus*, and this result corresponds with the heat diagram and PCA plots.

## 4 Conclusion

Pediatric febrile convulsions are common in children, with a secondary incidence of 30% to 40% after the first episode, mainly manifesting as sudden local or generalized myoclonic, compulsive convulsions.^9,10^ Repeated convulsive reoccurrences can cause irreversible brain damage in the child, in addition to the possibility of causing epilepsy.^11^ Lingzhu Pulvis,^12^ which is commonly used as a solid preparation in the treatment of pediatric febrile convulsions, has a high clinical use rate, but the volatile oil of *Acorus calamus* present in it is prone to oxidation instability and thus affects the efficacy of Lingzhu Pulvis.^13,14^ In this study, we introduced the Pickering emulsion technology based on the idea of “combination of medicine and adjuvant” to solve the defect of oxidation of the volatile oil of *Acorus calamus* in Lingzhu Pulvis^15^ and hence improve the stability and efficacy of Lingzhu Pulvis in the future. The final decision to use pearl powder as the stabilizer for Pickering was made by comparing the solubility, microstructure, emulsion type and zeta potential of a series of stabilizing agents. The best results were obtained with the sample prepared using a pearl powder concentration of 0.065 g mL^−1^, oil phase/water phase ratio 9 : 11 (v_1_/v_2_), and the high-pressure homogenization method. By measuring the malondialdehyde and peroxide contents in the volatile oils in each group, it was observed that the Pickering emulsion group showed a significant increase in antioxidant properties compared with the crude oil group. The analysis of the 25 chemical components showed that the Pickering emulsion group differed from the crude oil group in terms of better intra-group reproducibility and slower inter-group variability. This shows that the antioxidant property of the volatile oil of *Acorus calamus* was improved after the introduction of the Pickering emulsion technology, providing a theoretical basis for the next step in improving the stability of Lingzhu Pulvis and presenting a reference for the stability improvement of other-solid containing formulations.

## Consent for publication

All authors of the article have agreed to publish this paper.

## Data availability

The data and materials have been shown in the diagrams and attachments. If you need other materials, please send an email to the corresponding author.

## Author contributions

L. Peng and X. Zhang wrote the main manuscript text. D. Guo and B. Zhai analyzed the data. M. Wang, J. Zou and Y. Shi designed the study and all the authors amended the paper.

## Conflicts of interest

The authors declare that they have no conflicts of interest.

## Supplementary Material

RA-012-D2RA04433A-s001
